# Decomposition of Wavelength Dispersive X-Ray Spectra

**DOI:** 10.6028/jres.107.044

**Published:** 2002-12-01

**Authors:** Guy Rémond, Robert Myklebust, Michel Fialin, Clive Nockolds, Matthew Phillips, Claude Roques-Carmes

**Affiliations:** Australian Key Centre for Microscopy and Microanalysis, The University of Sydney, NSW 2006, Australia; Laboratoire de Microanalyse des Surfaces, Ecole Nationale de Mécanique et des Microtechniques, Besançon, France; National Institute of Standards and Technology, Gaithersburg, MD 20899-0001; Ura 736-CNRS, Université P et M Curie, Paris, France; Electron Microsco Unit, The University of Sydney, NSW 2006, Australia; Microstructural Analysis Unit, University Technology of Sydney, NSW 2007, Australia; Laboratoire de Microanalyse des Surfaces, Ecole Nationale de Mécanique et des Microtechniques, Besançon, France

**Keywords:** atomic lines, distortions induced by absorption edges, pseudo-Voigt profiles, satellites, soft x-ray bands, WDS instrumental distortions

## Abstract

Line shapes of atomic lines and soft x-ray emission bands measured with a wavelength dispersive spectrometer (WDS) with the Electron Probe Micro Analyzer (EPMA) are reviewed. Least square fitting to pseudo-Voigt profiles of the digitally measured spectra are used to account for the presence of non-diagram features (high and low energy satellites) and instrumental induced distortions. The effect of line width and relative intensities on the quality of fits is illustrated. Spectral distortions resulting from the presence of absorption edges within the analyzed wavelength region are illustrated for the case of FeLα,β emission bands for pure Fe and iron oxides. For quantitative analysis, an analytical approach is presented where the measured soft x-ray emission bands are corrected for self absorption before extracting the intensities from the experimental data.

## 1. Introduction

It is well recognized that the peak height of an x-ray emission line measured with a wavelength dispersive spectrometer (WDS) is a sufficient approximation for quantitative microanalysis on a routine basis using an Electron Probe Micro Analyzer (EPMA). This approach assumes that the observed x-ray line is symmetrical around the peak maximum occurring at a Bragg angle characteristic of the analyzed emission. A symmetrical peak never exists even for the case of atomic lines resulting from radiative transitions involving only core levels because of the presence of high and low energy satellites and instrumental distortions induced during measurement. While the approximation of the peak height for the x-ray intensity measurement remains valid in most analytical problems with the EPMA, this simplified approach is no longer sufficient in the presence of severe peak overlaps as is the case for L emission spectra of rare-earth elements [1] or when soft x-ray emissions are used in the analytical procedure [2–4].

We define soft x-ray emission as x-ray emission with energies lower than 1 keV such as the K emission series characteristic of low atomic number elements including carbon, oxygen, nitrogen, etc., and the L emission series chosen for intermediate atomic number elements. The soft x-ray emissions result from radiative transitions involving valence electrons. Consequently, the shape and the position of the maximum of soft emission bands are complex and depend on the electronic structure of the element within the matrix.

In the soft x-ray region, the peak height is no longer proportional to the peak area and several approaches have been proposed to determine the intensity of a soft x-ray emission band. For example, the use of predetermined “peak-to-area factor” as discussed by Bastin and Heijligers [5,6] or by measuring the peak area by summing the number of counts in each channel analyzed by stepping the monochromator across the wavelength domain containing the analyzed emission bands. The merits and limitations of these procedures have been discussed by Fialin et al. [7] accounting for the dependence of the peak shape on the self-absorption effect, the peak overlaps, and the resolution and the detection efficiency of the monochromator. However, it is still unclear whether the total area or only some spectroscopic features present in the measured spectra must be used to determine the intensity of the analyzed emission.

It is the aim of this paper to review the different features that lead to the complex shape of an x-ray line particularly within the soft x-ray emission domain. Practical considerations for WDS spectra processing using least-squares fitting techniques will be discussed. Applications to the interpretation of WDS spectra to the study of the chemical environment and quantitative microanalysis using soft x-ray emission bands will be illustrated using the FeLα,β emission bands measured from pure iron and iron oxides.

## 2. Contributions of X-Ray Generation Mechanisms and of Instrumental Factors to the Shape of a WDS X-Ray Line

### 2.1 The Diagram Lines

The energy loss due to inelastic scattering events produces a hole in the inner-shell of an ionized atom. The de-excitation processes leads to the emission of a mono-energetic photon which is characteristic of the atom. The energy of the emitted photon is equal to the energy difference Δ*E* of the energy levels involved in the radiative transition (in a non-radiative transition, the excess of energy Δ*E* contributes to the emission of an Auger electron).

The emitted photon is characterized by a Lorentzian energy distribution with a width at half maximum *Γ* (natural or physical width) related to the life time, τ, of the hole on the initial state according to:

Γτ=h/2π. (1)

The natural profile of a radiative transition is a convolution of energy distributions of each of the levels involved in the transition, which is a Lorentzian curve whose FWHM is equal to the sum of the FWHM of the two levels. Broadening may occur with low energy levels if non-radiative transitions are possible such as Coster-Kronig transitions. The natural profile for transitions involving valence electrons are also broader than those resulting only from core holes.

The probability *P*_if_ for a radiative transition between two levels i and f can be expressed according to:

$$P_{\text{if}}=\omega_{i}\;z_{if}\;N_{i}$$ (2)

where ***ω***_i_ is the fluorescence yield, *z_if_* is the weight of the line and *N*_i_ is the number of atoms in the initial level i per unit volume.

The fluorescence yield **ω** expresses the probability that the atom de-excites according to a radiative transition with the production of an x-ray photon. The probability to have a non-radiative transition with the emission of an Auger electron is (1−**ω**).

The fluorescence yield ***ω***_j_ of the level j is

$$\omega_{j}=N_{R}/(N_{R}+N_{\text{NR}})$$ (3)

where N_R_ and N_NR_ are the radiative and non-radiative transition rates, respectively.

For *n*_j_ ionisations created on level j, the number of photons in the j series is (***ω****_j_·n_j_*).

The intensity *I*_if_ in the radiative transition is proportional to *P*_if_ [Eq. 6)] and depends on the convolution of the initial *D*_i_ and final *D*_j_ energy level distributions:

$$I_{\text{if}}=P_{\text{if}}\;(D_{i}*D_{j})$$ (4)

For atomic lines involving only core levels, the convolution product *D*_i_ * *D*_j_ is assumed to be a constant. However, this approximation is no longer valid with soft (low energy) x-ray emissions since the final states are valence hole states so that the emission spectra will change with the electronic structure (density of occupied states, DOS) of the material. The position of the maximum energy and the intensity of the emission band will vary as a function of the chemical environment.

**Fig. 1:**
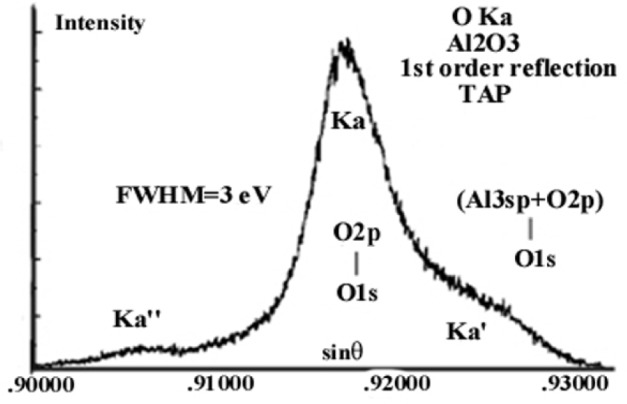
OKα emission band measured with a TAP monochromator from an alumina crystal consisting from the diagram peak associated with O_2_*_p_* –O_1_*_s_* transitions, a bonding peak associated with Al_3_*_sp_*,O_2_*_p_* –O_1_*_s_* transitions and a Ka″ high energy satellite associated either with multiple ionizations or instrumental distortion.

In wide band gap materials such as aluminum oxide, the major peak of a soft x-ray emission band (DOS) is usually accompanied by a low energy peak (“bonding peak”) resulting from transitions to the initial hole of electrons from mixing Al_3sp_ and O_2p_ energy states in the valence band, as illustrated in Fig. 1. The bonding peak resulting from the mixing of states (referred as Kα**′ in the literature) is located at approximately −5 eV from the maximum of the OKα parent peak. The feature labeled Kα″ in Fig. 1 occurring on the short wavelength (high energy) side of the diagram peak may result from satellite emissions (see Sec. 2.2) or from instrumental effects, as discussed below.

In wide band gap crystals, some high energy features may also result from transitions involving levels located in the band gap of the energy diagram of the crystal. These levels are associated with intrinsic point defects which are induced either during the crystal growth conditions or induced during the specimen preparation (polishing with abrasives) or by radiolysis mechanisms during the electron irradiation. In oxides, the most frequent defects are F^+^ and F centers, i.e., oxygen vacancies with one or two trapped electrons, respectively. As an exemple, Jonnard et al. [8] showed that the AlKβ emission (3*p* −1*s* transition) from alumina crystals is accompanied by a small high energy weak emission peak located 0.6 eV above the top of the valence band.

### 2.2 The Non-Diagram Lines

An x-ray emission line (or diagram line) resulting from a transition between two levels in the energy-level diagram is frequently accompanied by satellites (or non-diagram lines), i.e., x-ray lines whose energies do not correspond to the difference of two energy levels.

#### 2.2.1 High Energy Satellites

The high energy satellite lines have been intensively studied since the 1930s to 1940s beginning with the detailed works of Parratt [9,10] and Randall and Parratt [11]. Satellite lines result from electronic rearrangement concomitant with the ionization process during the de-excitation mechanisms of the ionized atoms.

##### K Lines

**Fig. 2:**
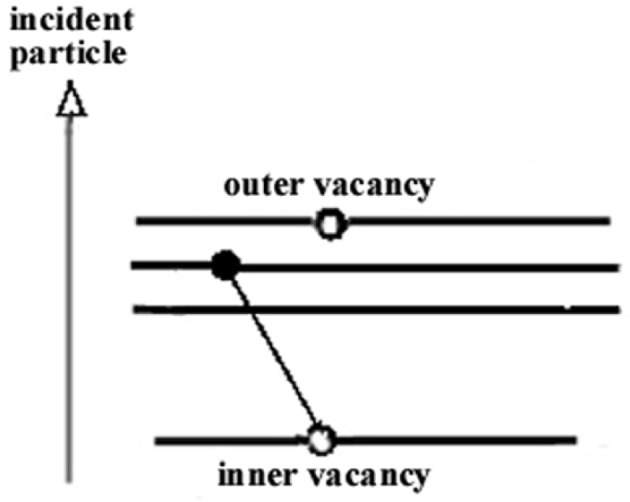
Emission of Kα_3,4_ high energy satellites.

When 1*s* and 2*p* vacancies are created simultaneously, the 2*p* vacancy has a relatively long life-time compared to that of the 1*s* vacancy. Thus, the inner vacancy de-excites in presence of a spectator hole which produces a change in the electrostatic potential leading to shifts in the energy levels (Fig. 2). The energy shifts for the Kα lines are given by:

$$\Delta E={\left(\Delta E\right)}_{1s}-{\left(\Delta E\right)}_{2p}.$$ (5)

The satellite lines resulting from the presence of outer vacancies consist of a number of closely spaced features. For the case of the Kα emission line, the high energy satellites are usually labeled as Kα_3,4_. The high energy satellite resulting from the de-excitation in presence of two outer vacancies is referred as Kα_5,6_ and exhibits a very weak amplitude. The energy separation distance between the satellite band and the Kα line ranges from about 10 eV up to about 40 eV for atomic number 12 < *Z* <30. Aberg [12] presents an extensive set of values for the relative intensity of the satellite which decreases from about 30 % for *Z* = 10 to 0.5 % at *Z* = 30.

##### L and M Lines

The de-excitation of an L or M level in presence of outer holes may also lead to the presence of high energy satellites associated with L or M x-ray peaks. The additional outer vacancies may result from Coster-Kronig transitions or shake-off mechanisms. The Coster-Kronig transitions result from an Auger process between sub-shells of the same shell.

The hole created on the L_1_ sub-shell may be filled by an electron originating from the L_2_ or L_3_ subshell. According to the selection rules, these transitions are not radiative and the excess of energy L_1_-L_2_, L_2_-L_3_ or L_1_-L_3_ is dissipated by the emission of an Auger electron from the M or N levels. The transition rate of non-radiative Coster-Kronig transitions *f*_ij_, where i and j are two subshells within the same energy level, is not permitted for all elements.

Indirect ionizations resulting from the non-radiative Coster-Kronig process have the following effects on the emission profile:

1. To create additional vacancies so that the total number of ionizations is the sum of the direct ionizations produced by the incident electrons and those created by the non-radiative Coster-Kronig transition. For example the Lα emission line involving ionization on the L_3_ subshell, the number of Lα photons will be:
  $$n_{L_{3}}<\omega_{L_{3}}>\;=\omega_{L_{3}}\;[n_{L_{3}}+f_{13}n_{L_{1}}+f_{23}(n_{L_{2}}+f_{12}n_{L_{1}})]$$ (6)
  where *f*_13_, *f*_23_, and *f*_12_ are the Coster-Kronig transition probabilities.
2. To leave outer vacancies during the re-arrangement of the ionized states between the sub-shells prior to the radiative transition with the emission of an x-ray photon. This process is responsible for the production of high energy satellites. When the atomic number of the emitter decreases, the energy separation distance between the shake-off satellites and the diagram also decreases and may be observed as a shoulder to the major peak.

According to Fabian [13], several line shapes of L emission spectra of elements in the first transition series can be distinguished depending on the incident electron energy region: 1) *The Threshold Excitation Region* when the incident energy lies between the L_3_ and L_2_ energy thresholds, multiple vacancy satellites are largely reduced, 2) *The Satellite Region* in which the Lα diagram line becomes distorted by the progressive development of high energy satellites when the incident electron energy increases from the L_3_ sub-shell threshold up to about three times that value, and 3) *The Self Absorption Region* for incident energies greater than about 3 or 4 times the L_3_ threshold energy, the fine structure vanishes and the effect is attributed to self-absorption. Increasing incident electron energy also increases the absorption path of the generated x-ray photons within the specimen and self-absorption removes the fine structure when a high incident energy is used.

**Fig. 3:**
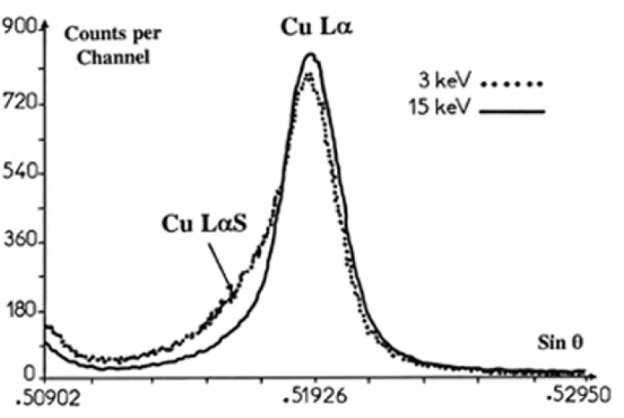
CuLα line shape changes as a function of the incident energy. Note that the L_3_ absorption edge lies between the main diagram peak and the high energy satellite.

Peak shape changes as a function of the incident energy is illustrated in Fig. 3 for the case of the CuLα emission from pure copper measured with a TAP monochromator. The variation of the intensity of the high energy satellite relative to that of the diagram peak as a function of the beam energy results from a differential self absorption effect because the L_3_ absorption edge occurs between the two spectral features.

Similarly, high energy satellites associated with Mα lines of elements with high atomic number result from the M_5_ hole de-excitation in presence of simultaneous vacancies in the M_5_ and N sub-shells. Only the envelope of satellites resulting from additional vacancies in the M_5_ sub-shell can be distinguished from the diagram line.

**Fig. 4:**
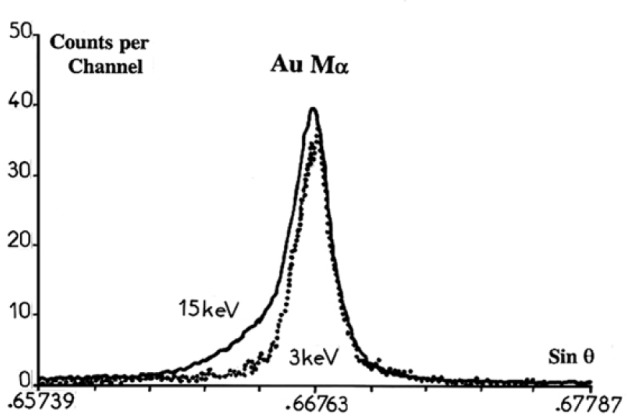
Line shape changes of the AuMα emission peak as a function of the incident energy. The 3 keV incident energy is lower than the M_1_ and M_2_ ionization thresholds.

As shown in Fig. 4, peak shape changes as a function of the beam energy are also observed for the case of the AuMα emission band measured from a pure Au specimen with a PET monochromator at 3 keV and 15 keV successively. The excitation energy thresholds for the M_1_ to M_5_ sub-shells are 3.425 keV (M_1_), 3.150 keV (M_2_), 2.743 keV (M_3_), 2.291 keV (M_4_) and 20.206 keV (M_5_), respectively. Thus, a 3 keV incident energy is sufficient to provoke the Au Mα emission involving the initial hole in the M_5_ sub-shell. Additional vacancies may be created in the M_3_ and M_4_ sub-shells with possible outer N vacancies resulting from Coster-Kronig transfer of the type M_3,4_–M_5_N_x_, producing weak high energy satellites. No additional vacancies are created in the M_1_ and M_2_ levels and transfer of the type M_1,2_–M_5_N_x_ does not exist for a 3 keV incident energy. Reciprocally, the M_1_ and M_2_ sub-shells are excited with a 15 keV incident energy and the resulting inner vacancies can move to the M_5_ sub-shell with production of outer holes by Coster-Kronig mechanisms, thus the pronounced asymmetry on the high energy side of the Au Mα may reasonably be assigned to the development of satellites resulting from the de-excitation of the M_5_ level in presence of outer N vacancies.

#### 2.2.2 Low Energy Satellites

Several theories are available to describe the Kβ**′ low energy feature associated with the Kβ_1,3_ emission resulting from transitions involving the partially filled 3d shells of transition elements and their oxides.

**Fig. 5:**
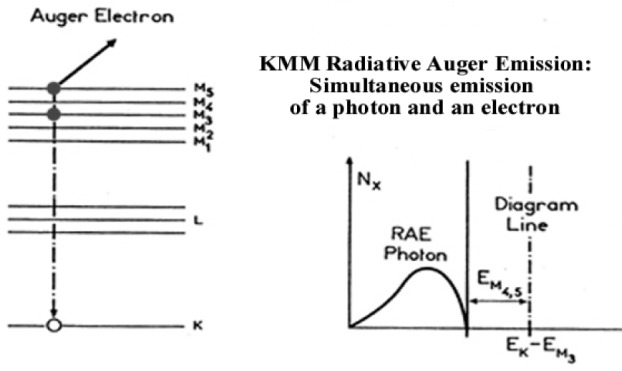
Low energy satellite resulting from Radiative Auger Emission.

The Radiative Auger Effect (RAE) produces a broad structure at a lower energy than the characteristic diagram line. The RAE process results from a de-excitation of a K vacancy, similar to an Auger process with simultaneous emission of a bound electron and an x-ray photon (Fig. 5). For atomic number 15 < *Z* < 30, the low energy structures associated with the Kβ_1,3_ diagram line, can be interpreted in terms of KMM radiative Auger effect Radiative Auger Emission [14].

According to Salem et al. [15], the interaction between the electrons in the incomplete 3d shell and the hole in the incomplete 3*p* shell splits both 3*p* and 3*d* levels causing a demultiplication of transitions.

The Kβ**′ satellite has also been explained in terms of the plasmon oscillation theory [16]. During the x-ray emission process, the transition valence electron excites a plasmon in the valence band. The transition energy of the Kβ_1,3_ line will thus be shared between the plasmon and the emitting photon which will be deprived of an energy equal to the plasmon energy. For the transition elements the energy separation distance between the Kβ**′ satellite and Kβ_1,3_ diagram line is in the order of magnitude of 10 eV, depending upon the chemical environment [16].

**Fig. 6:**
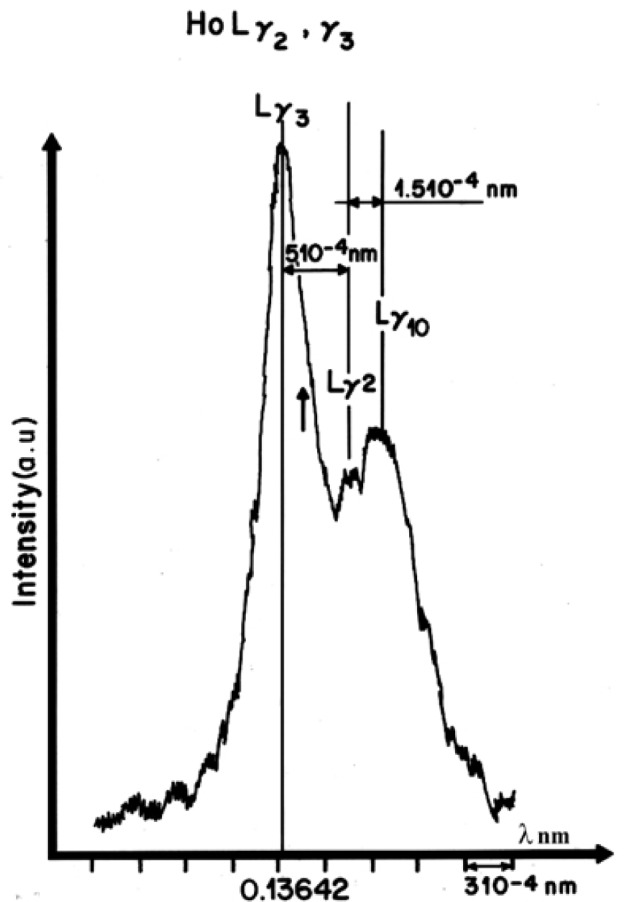
Low energy satellite HoLγ_10_ associated with the HoLγ_2,3_ emission lines measured with a Johansson mounted quartz monochromator.

The theories concerning the production of Kβ**′ satellite associated with the Kβ_1,3_ line of transition elements were extended to the case of the L x-ray spectra of the lanthanide elements [17]. The Lβ_2_, Lβ_4_, Lγ_1_ and Lγ_2_ emissions exhibiting low energy effects are associated with transitions involving the partially filled 4*f* shell. The energy separation distance between the low energy satellite and its parent line is a few tens of eV and are easily detected with the resolution of the WDS of the EPMA as illustrated in Fig. 6, for the low energy (long wavelength) satellite labeled Lγ_10_ associated to the HoLγ_2,3_ peaks measured with a fully focusing quartz monochromator (Johannson mounting).

In practice, only the convolution of these features with the spectral window (or energy response function) of the spectrometer will be seen. Thus the ability to observe the non-diagram satellite bands will depend on the resolution and sensitivity of the spectrometer as reported by Rémond et al. [4,18] and Fialin et al. [2,3] for x-ray emission spectra measured with EPMA’s equipped with WDS.

### 2.3 Instrumental Distortions

Modern EPMAs are generally equipped with no-slit spectrometers in which the monochromator is a crystal bent to yield a concave cylindrical surface producing a point focus image of the point x-ray point source. In a symmetrical system, the curved Bragg planes are parallel to the crystal surface and the incident and “reflected” rays are located on the same side of the monochromator surface.

According to the Johann mounting, a flat crystal is cylindrically bent to twice the focal circle radius so that the focusing conditions are only satisfied for x-ray beams incident at the “center” of the monochromator, i.e., the point where the focal circle is tangent to the crystal surface. A deviation from the focusing conditions will increase the further the incident x-ray beam is from the center of the monochromator (semi-focusing geometry). Away from the center of the crystal the small distance between its surface and the focal circle will give rise to a focusing defect, producing a broadening and a decrease in intensity of the observed x-ray peak According to Cauchois and Bonnelle [19], the line width due to departure from the Bragg conditions in the median plane of the crystal is given by:

ΔL=(ϑ2/8R)cotθ (7)

where ϑ is the linear opening of the crystal and *R* the focal circle radius.

**Fig. 7:**
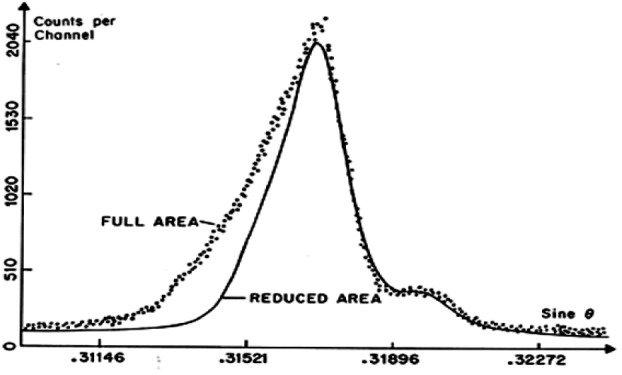
Line shape of the AuLα peak analyzed using the full area of a LiF monochromator and after reducing the active area of the same monochromator.

Reducing the linear opening will minimize the focusing defect resulting from departure from point-to-point focusing conditions (point source and point image being both located on the focal circle) when a Johann mounted bent crystal is used as shown in Fig. 7 for the Au Lα emission line measured with a Johann mounted LiF monochromator. The measurements were successively performed using the full area of the monochromator and after its active area was reduced by covering the edges of the crystal with narrow bands of lead. (Rémond et al. [18]).

The line broadening for rays far from the center of the crystal cancels for a curved crystal of the Johansson type, i.e., when the Bragg focusing conditions are satisfied for all x-rays impinging the crystal surface. According to this mounting set-up, the crystal planes are bent to a radius of curvature 2*R* and the surface of the crystal is ground to a circle radius *R*. With this geometry, the entire surface of the crystal is tangent to the focusing circle and the Bragg conditions are satisfied for all points at the monochromator surface (fully focusing geometry).

The observed peak profile and intensity depend on the reflectance coefficient, *R*(*θ*), of the crystal for the direction *θ* with :

$$R(\theta )=I_{R}/I_{0}$$ (8)

where *I*_R_ and *I*_0_ are the intensities of the reflected and incident x-ray beams, respectively. The graph of *R* (*θ*) as a function of *θ* is the diffraction pattern of the crystal. The reflecting power *P*, is given by

P=∫R(θ)dθ. (9)

The reflectance coefficient is a function of the refractive index. For x-ray frequencies, the refractive index <*n*> is complex:

<n>=n−iβ (10)

The real part of the refractive index, *n*, is slightly lower than unity and the decrement *δ*= 1 – *n* characterizes the dispersion. The imaginary part, *β*, of the refractive index is the extinction coefficient. It is related to the ordinary linear absorption coefficient μ by :

β=(λ/4π)μ. (11)

The refractive index is complex, the extinction coefficient introduces a decrease of the amplitude of the waves passing through the crystal and phase changes between the incident and successively reflected waves

Neglecting the absorption due to the photoelectric effect and incoherent scattering, Darwin [20] showed that the reflection from a perfect plane crystal should be total over a narrow angular range, ***Ω***, outside this range the reflection diminishes rapidly and symmetrically. However, the diffraction pattern of a perfect plane crystal, lies below the Darwin curve and has an asymmetrical shape due to absorption resulting from the complex nature of the refractive index, i.e., decrease of the amplitude and phase changes between the waves.

For a crystal bent with a long radius of curvature, the reflectance curve remains very similar to the Darwin band of a flat monochromator. For short radius of curvature, the angle of reflection will change appreciably as a function of depth below the crystal surface leading to an exponentially decreasing tail on the low Bragg angle side.

**Fig. 8:**
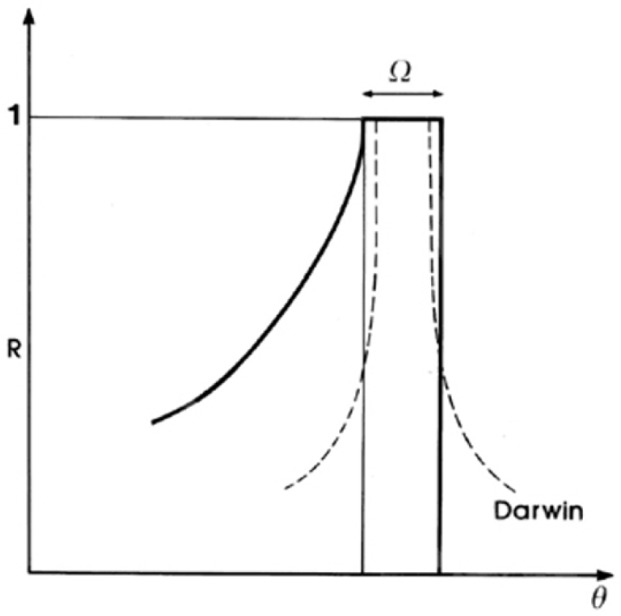
Diffraction pattern of a bent monochromator.

Cauchois and Bonnelle [19] showed that the resulting Darwin curve for the bent crystal is made-up of adjacent rectangles of decreasing height as shown schematically in Fig. 8.

The line width, Δ*L*, arises from the intrinsic nature of multiple reflections inside the monochromator. According to Cauchois and Bonnelle, [19] the line width, Δ*L*, along the focal circle, i.e., for rays incident near the center of the crystal so that the source and its image are on the same focal circle of radius R is:

ΔL=RΩ+(cosθ/2μ)ln2. (12)

The first term, *R****Ω***, corresponds to a symmetrical contribution to the shape of the observed line and the second term, (cos*θ*/2*μ*) ln2, expresses an asymmetrical tail occurring on the short wavelength side of the peak

At high Bragg angles (θ≫ 35°) or for high absorption of the incident photons, only the outer surface of the crystal reflects the beam and the crystal behaves as a perfect crystal and second term in Eq. (12) is negligible. However, at low Bragg angles or for high energy rays penetrating deep in the crystal, this term is no longer negligible and an asymmetric diffraction pattern is found.

**Fig. 9:**
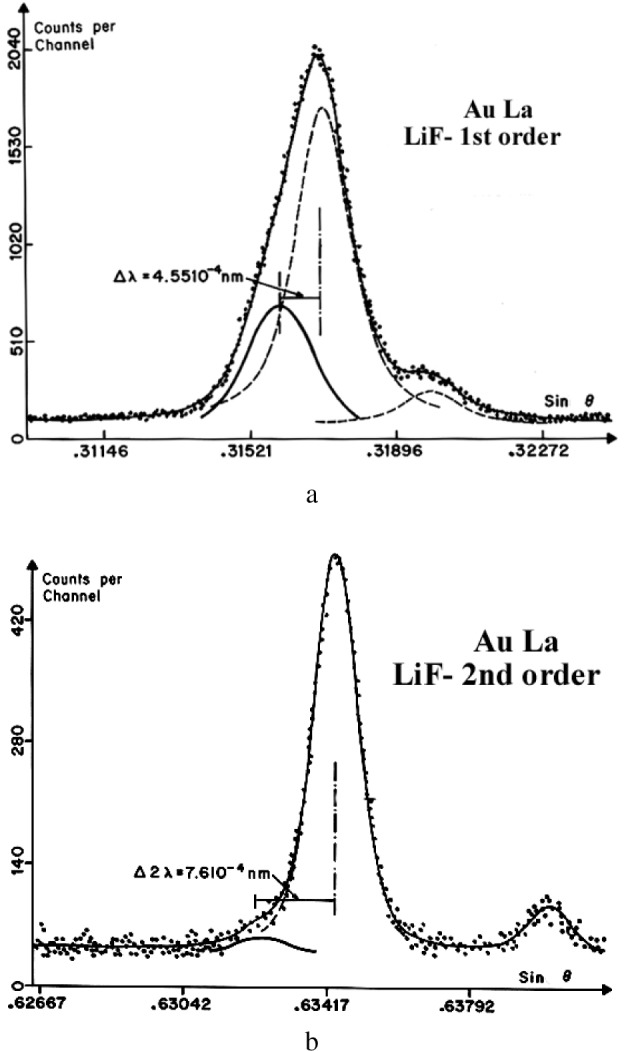
Instrumental distortion of x-ray lines occurring at low Bragg angles. (a) first order reflection AuLα peak measured with a LiF monochromator (17°50″ Bragg angle) and (b) second order reflection.

The effect of the Bragg angle on the shape of an x-ray peak is illustrated in Figs. 9a,b for the first and second order reflection of the AuLα emission measured with a Johann mounted LiF monochromator.

**Fig. 10:**
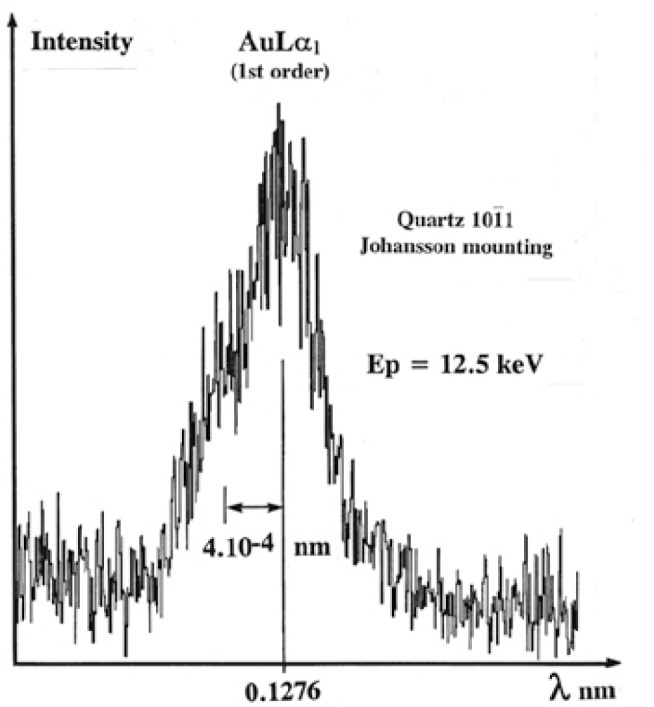
First order reflection of the AuLα peak analyzed with a quartz monochromator (Johansson mounting) and a 12.5 keV incident energy just above the AuL_3_ excitation threshold.

The broadening due to the thickness of the crystal will affect all incident rays impinging upon the surface along the focal circle and the Δ*L* broadening effect will occur either for Johann or Johansson curved crystals. The focusing defect resulting from the thickness of the crystal is illustrated in Fig. 10 for the AuLα peak measured with a quartz monochromator installed according to the Johansson mounting. For this measurement, the incident energy was set at 12.5 keV, a value just above the L_3_ excitation threshold but lower than that of the L_1_ and L_2_ levels. Under these conditions, the Coster-Kronig transitions are not produced and the tail observed on the short wavelength side of the AuLα peak must be assigned to an instrumental defect rather than a high energy satellite.

In some instances, artifacts may result fom the interactions of the incident x-ray photons with the monochromator.

For example, it may be difficult to identify with certainty the Ka**′′ band shown in Fig. 1 with the presence of a high energy satellite due to the OKα emission resulting from de-excitation in presence of outer vacancies because an anomalous reflectivity of the monochromator containing oxygen may also lead to the presence of a parasite band. As reported by Mattson and Ehlert [21], the weight of the instrumental Ka**′′ artifact to the OKa main peak is about 50 % when measured with a KAP monochromator and is considerably reduced when measured with a TAP monochromator.

Another example of artifacts encountered in WDS is the presence of a “hole” in the continuous emission distribution as reported by Self et al. [22]. For a symmetrical reflection geometry using a bent monochromator, reflection of the x-ray beams by crystallographic planes not parallel to the crystal surface may occur. In a single crystal, diffraction can occur from any atomic planes

The monochromator is assumed to be bent along the (*hkl*) planes. If an x-ray beam makes an angle, *θ*, with the planes and an angle, *θ*′, with the (*h'k'l*') planes, the same wavelength will be diffracted by the two sets of planes when :

$$2d\;(hkl)\;\sin\theta =2d^{\prime }\;(h^{\prime }k^{\prime }l^{\prime })\;\sin\theta^{\prime }.$$ (13)

In this situation, diffraction by the (*h'k'l*') plane will cause a decrease in the intensity of the primary beam to be diffracted by the (*hkl*) planes.

The presence of the “hole” in the intensity distribution of the continuous emission can be related to multiple reflections on planes differently orientated below the monochromator surface. Self et al. [22] calculated the positions of “holes” occurring at specific wavelengths in the continuous spectrum by considering diffraction from crystallographic planes different from the (200) planes of the LiF monochromator.

**Fig. 11:**
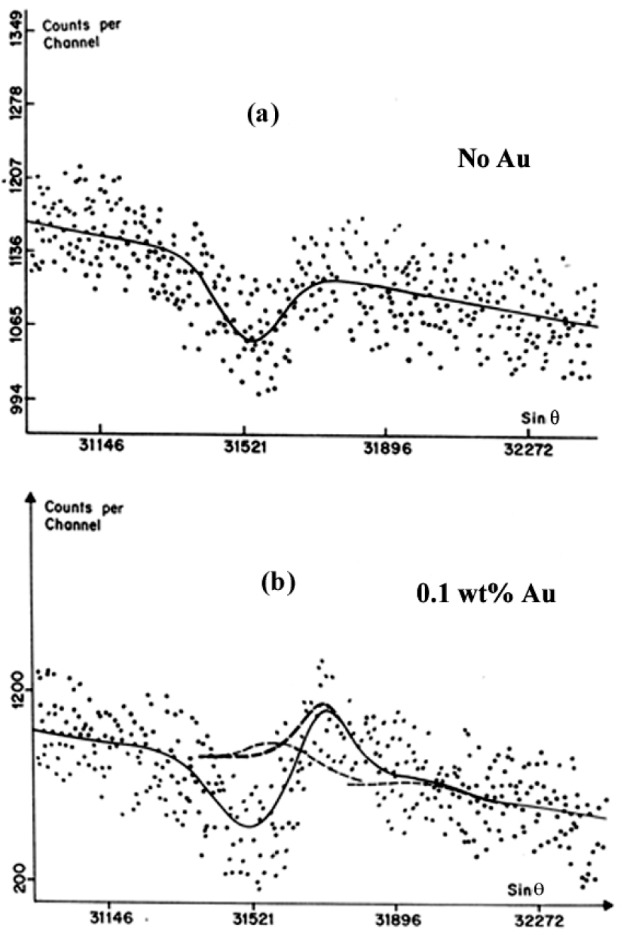
Effect of instrumental distortions on the observed AuLα line analyzed with a LiF monochromator. (a) decrease of the continuous emission intensity resulting from multiple reflections on planes differently orientated below the surface monochromator, (b) observed AuLα peak for gold present at trace level resulting from the presence of the hole in the continuum emission and the instrumental distortion due to focusing defect of the monochromator as shown in Fig. 9a.

The simultaneous contribution of the hole in the continuous emission and the instrumental distortion due to the thickness of the crystal is illustrated in Fig. 11 for the AuLα peak characteristic of Au present at trace level in an arsenopyrite (AsFeS) specimen. This example clearly illustrates the need for spectral decomposition of the observed peak in order to derive accurate intensities as previously discussed by Rémond et al. [18,23].

## 3. Deconvolution vs Spectral Decomposition Using Least-Square Fitting Techniques

The observed photon distribution *P*(*E*) within an x-ray emission peak is expressed as:

$$P(E)=\int L(E^{\prime })\;F\;(E-E^{\prime })\;dE^{\prime }$$ (14)

where *L*(*E*) is the physical photon energy distribution, *F*(*E*) is the instrumental response function, *E*' is the energy of the x-ray radiation and *E* is the energy at the center of the peak.

Equation (14) can only be solved if the *L*(*E*) and *F*(*E*) distributions are known for all analysed photon energies. The natural width of an x-ray emission is generally well described according to a Lorentzian distribution

$$L(E)=\frac{H}{1+{\left[(E-E_{0})/\gamma \right]}^{2}}$$ (15)

where γ is the half-width at half maximum (HWHM), H is the amplitude of the distribution centered at energy *E*_0_. The response function *F*(*E*') expresses the observed line shape of a mono-energetic photon assumed to have a natural width equal to zero (or negligible) with respect to the energy resolution of the spectrometer.

In practice, for quantitative analysis with the EPMA, a full deconvolution procedure is not required and the data processing only aims to measure the intensities of partly or fully overlapping components in the observed spectrum. Instead of deconvolution procedure in the strict sense based on Eq. (24), spectral decomposition based on least square fitting techniques of the measured spectrum to analytical descriptions of x-ray peaks is more frequently used in EDS and WDS quantitative x-ray analysis. The fitting function describing the shape of the observed peak includes the peak position, the peak intensity and the peak width as variables. In an analytical description of the peak shape only an effective width representing the combination of the natural and instrumental contributions is used.

### 3.1 A Need for a Unique Approach for EDS and WDS Spectra Processing

**Fig. 12:**
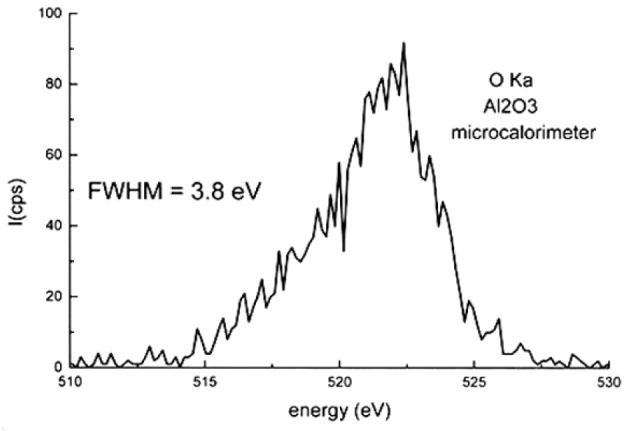
OKα from an alumina specimen measured with the microcalorimeter (spectrum measured by Dr. Wollman at NIST).

The energy resolution of an EDS spectrum obtained with a microcalorimeter [24] is similar to that obtained with a WDS as illustrated in Fig. 12 for the OKα emission band from an Al_2_O_3_ specimen. Therefore, there is thus a need to use a unique analytical description of the shape of an x-ray line to least-squares fit to the WDS and high resolution EDS spectra.

Studying the response of solid-state energy dispersive detectors to high energy mono-energetic incident radiations Phillips and Marlow [25] expressed the observed line shape *P*(*E*) as a function of the analyzed photon energy *E*, according to:

P(E)=S(E)+D(E)+G(E) (16)

The above expression is known as the Hypermet function in which *S*(*E*) represents the Compton scattering of photons within the detector, *D*(*E*) expresses the phenomena of incomplete charge collection in the dead layer of the solid-state detector and *G*(*E*) is the major Gaussian peak whose the width is large with respect to the intrinsic width of the diagram line.

According to the Hypermet function, the asymmetry of peaks resulting from the photon-detector interactions is treated by adding two analytical expressions *S*(*E*) and *D*(*E*) to that describing the spectroscopic features. Several expressions for *S*(*E*) and *D*(*E*) are available depending upon the analyzed photon energy domain and the type of detectors as discussed by Campbell et al. [26].

It is widely accepted that describing a measured x-ray peak by a Gaussian distribution is a sufficient approximation to derive accurate x-ray intensities from an x-ray emission spectrum measured with an EDS. Most software available with commercial EDS systems use a Gaussian approximation for describing the observed shape of an x-ray peak which is not distorted by instrumental factors. The incomplete charge collection phenomenon *D*(*E*) in the dead layer of the detector, dominates the asymmetry of low energy x-ray peaks analyzed by means of a Si(Li) detector.

### 3.2 The Fitting Function to Mono-Energetic Features

The observed shape of an x-ray line is controlled by the intrinsic properties of the spectrometer and its geometrical arrangement within the specimen chamber. Thus, depending on the resolution of the spectrometer, there is no evidence that the line shape function satisfies a purely Gaussian or a purely Lorentzian distribution function.

A pseudo-Voigt function, *P*(λ), can be used as a fitting function to the measured WDS peak shape [18,27] according to:

P(λ)=CgG(λ)+1L(λ) (17)

where 0 ≤ *C*g ≤ 1 and *C*l = 1 – *C*g are the contributions of the Gaussian *G*(*E*) and Lorentzian *L*(*E*) of same width (*Γ*g = *Γ*l) and centered at the same position. Each function in the linear combination shown in Eq. (17) is weighted by a coefficient *C*g expressing the intermediate nature between Gaussian and Lorentzian shaped WDS x-ray peaks.

### 3.3 The Fitting Function to the Continuous Emission Distribution

Spectral decomposition of either EDS or WDS characteristic x-ray emission peaks can only be applied when the underlying continuous emission has been removed or when an analytical description of this emission has been added to the fitting procedure.

When a full WDS spectrum is measured it is necessary to account for the variations of the absorption as a function of wavelength as is the case when modeling the continuous emission for an EDS spectrum. For this purpose, a physical description of the WDS continuous emission should be used such as the model proposed by Smith and Reed [28] for the description of the continuous emission associated with WDS spectra of rare-earth bearing compounds.

When the absorption edge is located within the narrow wavelength domain containing the emission band, for example the L emission spectra of transition elements, it is necessary to correct the observed peak shape for self-absorption before extracting the peak intensities.

According to Fabian [13], a self-absorption spectrum can be obtained from two spectra measured from two different excitation conditions. The method consists in normalizing the two spectra and then dividing them channel by channel.

The observed spectrum is the sum of the number of counts *I*_i_ in each channel i within the wavelength domain containing the line of interest. Let *Ī*_i_, be the intensity *I*_i_ at channel i, normalized to that *I*_0_ at the peak maximum of the analyzed line.

Let us assume that the incident energy *E*_1_ is low enough to neglect the absorption effect. Thus, *Ī*_i_(*E*_1_) represents the generated intensity in channel i of a distribution whose the maximum intensity is equal to unity:

$${\bar{I}}_{i}(E_{1})=I_{i}(E_{1})/I_{0}(E_{1})=I_{i}(E_{1})/[Z(E_{1})]$$ (18)

where *I*_0_(*E*_1_) = ∫ φ(*ρz*) d*ρz* = [*Z*(*E*_1_)] is the atomic number correction factor since the absorption for *E*_1_ is negligible.

For an incident energy *E*^2^ > *E*_1_, the absorption effect is no longer negligible and *Ī*_i_(*E*_2_) is the emitted intensity, in channel i, of a distribution whose the maximum intensity is equal to unity expressed by:

$${\bar{I}}_{i}(E_{2})=I_{i}(E_{2})/I_{0}(E_{2})=I_{i}(E_{2})/[Z(E_{2})]f(\chi_{0})]$$ (19)

since for the energy *E* (_2_) we have *I*_0_(*E*_2_) = ∫ φ(*ρz*)exp(−*χρz*) d*ρz* = [*Z*(*E*_2_)] *f* (*χ*_0_), where *f* (χ_0_) is the usual absorption correction factor for the wavelength corresponding to the peak maximum.

The intensity ratio between the two normalized spectra *Ī*_i_(*E*_1_) and *Ī*_i_(*E*_2_) measured at low (*E*p2_1_) and high (*E*_2_) incident energy successively is thus:

$$g(\chi_{i})={\bar{I}}_{i}(E_{1})/{\bar{I}}_{i}(E_{2})$$ (20)

**Fig. 13:**
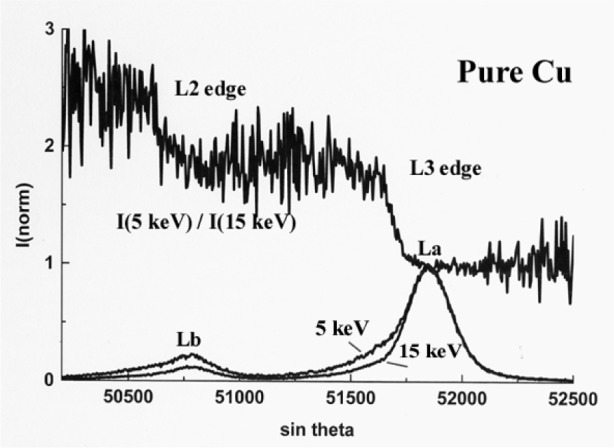
Self-absorption correction factor for CuLα in pure Cu derived from the intensity ratio of two CuLα spectra measured at 3 keV and 15 keV incident energy successively.

The graph *g*(χ_i_) of the ratio of the normalized intensities as a function of wavelength (or channel i) expresses the variations of the absorption correction factor relative to that at the peak maximum accounting for the presence of the absorption edges in the analyzed wavelength region as illustrated in Fig. 13 for the case of the CuLα,β emission spectra.

Since the two normalized spectra have the same amplitude at their maximum, the intensities *Ī*_i_(*E*_1_) and *Ī*_i_(*E*_2_) represent the fraction of generated and emitted intensities respectively with respect to the maximum intensity equal to unity. Consequently, *g*(*χ*_i_) is an equivalent absorption correction factor in channel i, relative to that for the channel corresponging to the peak maximum.

Combining Eqs. (18), (19) and (20) leads to:

$${I^{i}}_{\text{gen}}={I^{i}}_{\text{mea}}*g(\chi_{i})*\frac{\left[Z(E_{1})\right]}{\left[Z(E_{2})]f(\chi_{0}\right)}$$ (21)

where *I*^i^_gen_ is the calculated generated intensity corresponding to the measured intensity *I*^i^_mea_, *f*(*χ*_0_) is the normal absorption correction factor for the wavelength corresponding to the peak maximum and [Z] designates the atomic correction factor. The graph *g*(*χ*_i_) is determined empirically from two measurements performed at low and high incident energy successively and the scaling factor $\frac{\left[Z(E_{1})\right]}{\left[Z(E_{2})]f(\chi_{0}\right)}$ is calculated using Monte-Carlo calculations or by means of the analytical expressions commonly used for quantitative x-ray microanalysis.

A second approach for deriving the variation in the *f*(*χ*) correction factor within the analyzed wavelength domain containing an absorption edge has been previously discussed by Rémond et al. [5]. The approach consists in transferring the WDS spectrum into the energy space and to model the continuous emission *N*(*E*) as a function of the energy according to:

$$N(E)=Z\left[a\left(\frac{E_{0}-E}{E}\right)+b{\left(\frac{E_{0}-E}{E}\right)}^{2}\right]f(E)D(E)$$ (22)

where *E*_0_ is the incident energy, *E* the current photon energy, *f*(*E*) is the absorption correction factor at energy *E* and *D*(*E*) is the detection efficiency.

**Fig. 14:**
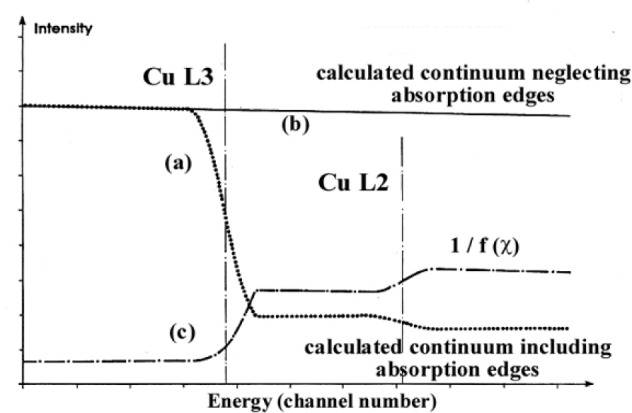
Continuous emission distribution as a function of photon energies associated with the CuLα,β emission bands for a pure copper specimen. (a) calculation accounting for the absorption edges, (b) calculations neglecting the absorption edges, and (c) absorption correction factors given by the ratio of the two calculated (a) and (b) continuous emission distributions.

The continuous emission is calculated taking into account the presence of the absorption edges and then adjusted to the experimental spectrum. The calculation is repeated by omitting the presence of the absorption edges, i.e., assuming a continuous linear variation of the continuum in the analyzed energy domain. The ratio of the two calculated curves gives the correction factor to be applied to each channel as illustrated in Fig. 14 for the CuLα,β EDS spectrum derived from the experimental WDS data.

When the normalized absorption correction factors for each channel are obtained, it is possible to reconstruct the generated emission spectrum. Dividing the real value of each channel of an experimental spectrum measured with the high incident energy by the corresponding channel in the graph of the correction factor provides a spectrum whose the shape is corrected for the differential absorption within the analyzed wavelength domain. The corrected spectrum corresponds to an experimental spectrum superimposed on a constant linear continuum emission intensity within the wavelength domain containing the emission bands of interest. The generated emission spectrum is thus obtained by multiplying the content of each channel of the spectrum corrected for the presence of the absorption edges by the calculated value of the generated intensity at the peak maximum. That value is derived from the measured peak height intensity of the Lα line using usual ZAF or *φ*(*ρz*) procedures with the absorption coefficient for the mono-energetic Lα line obtained from data tables.

## 4. Applications of Least-Square Fitting Techniques to WDS Spectra

### 4.1 Practical Considerations

The WDS x-ray peaks were digitally recorded by moving the monochromator step by step. Before displaying the x-ray peaks, the measured intensity in each channel was corrected for dead time.

For each analyzed peak the fitting procedure was conducted using a set of pseudo-Voigt profile with additional Gaussian or pseudo-Voigt offsets and except when specified, the intensity of the continuous emission underlying the analyzed x-ray peaks was approximated by a linear function.

The adjustment of the fit of the experimental spectra to the model function is done with an interactive multiple least square fitting program developed by Massiot [29], minimizing the residual distance normalized to the number of data points (standard deviation SD) according to

$$SD={\left(\sum \frac{{\left[P^{1}(\lambda_{i})-P^{1}(\lambda_{i})\right]}^{2}}{n}\right)}^{1/2}$$ (23)

where *n* is the number of data points, *P*′(*λ*i) is the number of counts at channel i and *P*′(*λ*i) is the value of the model function at that point i. The method utilizes an iterative non-linear least-squares fitting process starting from an initial estimated solution.

The shape of a peak resulting from the simultaneous presence of a diagram and non-diagram bands can be described by the sum of analytical pseudo-Voigt functions which describe each of the spectral components.

In previous studies, Rémond et al. [4,18] added Gaussian or pseudo-Voigt (equation [17] offsets to the main profiles to describe the high energy tail of x-ray emission peaks occurring at low Bragg angles. This approach based on the addition of high energy offsets, is supported by the results by Cauchois and Bonnelle [19] who showed that the asymmetrical diffraction pattern for a bent monochromator can be approximated by a sum of adjacent rectangular Darwin curves with decreasing amplitudes.

An observed x-ray peak will be described as the sum of many pseudo-Voigt profiles, each of them being characterized by four parameters, the proportion *C*g of Gaussian to Lorentzian distribution, the peak position, the peak width and the peak amplitude. With the available least-squares fitting program used, the parameter *C*g must be empirically determined and used as a constant in the fit, all other parameters being kept as variables. When possible, some of the variables must be predetermined and used as constants or be coupled to each other in order to impose physical constraints and to reduce the number of variables.

### 4.2 Gaussian to Lorentian Proportion and Line Widths

As previously illustrated in Ref. [18], the shapes of the SKα NbLα and AuMα diagram peaks of similar energy were analyzed with the PET monochromator and were found to be very different. The proportion of Gaussian and Lorentzian distributions in the peak profile is a function of the intrinsic properties of the analyzed emission, i.e., of the energy sub-shells involved in the analyzed radiative transitions.

In practice, the value of the *C*g parameter in Eq. (17) is determined by varying step by step the *C*g value from 0 (pure Lorentzian) to 1 (pure Gaussian) until a satisfactorily description of the long wavelength side of the peak is obtained. This approach assumes the absence of low energy satellites such as RAE satellites, which usually have weak amplitudes. This approach is more difficult to apply to unresolved α_1_–α_2_ doublets or to soft x-ray emission bands since the bonding peak occurs on the low energy side of the diagram band as previously illustrated in Fig. 1. In the presence of overlapping peaks a data base is required to couple the peak positions and amplitudes in order to accurately determine the low energy peak profile.

In order to reduce the number of variables in the fit, a solution includes the determination of a calibration curve for the peak widths as a function of the wavelength domain analyzed by each monochromator. Such calibration curves should be useful to generate synthetic reference spectra.

In practice, specific calibration curves of peak widths as a function of wavelength must be obtained for emission lines belonging to the K, L, or M series since these lines of similar energy do not only exhibit shape changes but also have different peak widths [4]. These variations account for the contribution of the natural width to the observed peak width. Rémond et al. [1,18] used an approach where the observed width, used as a variable in the fitting function, was replaced by quadratic addition of the instrumental resolution *Γ*_i_ and natural width γ, according to:

$$\Gamma ={\left[\Gamma_{i}^{2}+\gamma^{2}\right]}^{1/2}$$ (24)

**Fig. 15:**
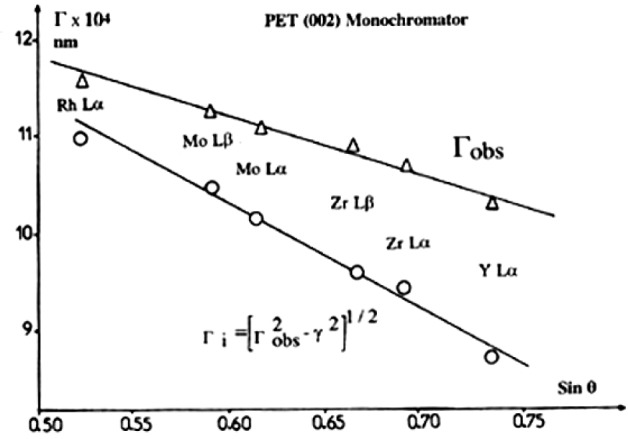
Calculated FWHM and instrumental response for function for x-ray lines analyzed with a PET monochromator.

The width of Lα and Lβ_1_ peaks of pure elements analyzed with a PET monochromator (Bragg angles greater than ≈30°) exhibited a linear variation as a function of the analyzed wavelengths as shown in Fig. 15. From all analyzed L x-rays peaks, the resolution *Γ*_i_ of the spectrometer equipped with a PET monochromator was calculated using Eq. (22). As shown in Fig. 15, a linear relationship was found to exist between the analyzed L emission peak and the instrumental resolution *Γ*_i_. This relation can be used to determine the width of any peak in the analyzed wavelength domain and can be used as a constant in the fitting procedure rather than a variable. The validity of the simplified approach in Eq. (22) was supported by comparing observed and calculated widths of the NbLα (2196 eV) and AuMα (2123 eV) lines measured with the PET monochromator [18].

The calculated width of the experimental NbLα peak was found to be 4.1 eV, which differs by less than 3 % of the observed peak width of 3.97 eV. Similarly, the calculated intrinsic width for the AuMα line was found to be 2.7 eV, which is very close to intrinsic width at 2.5 eV reported by Laakkonen and Graeffe [30].

**Fig. 16:**
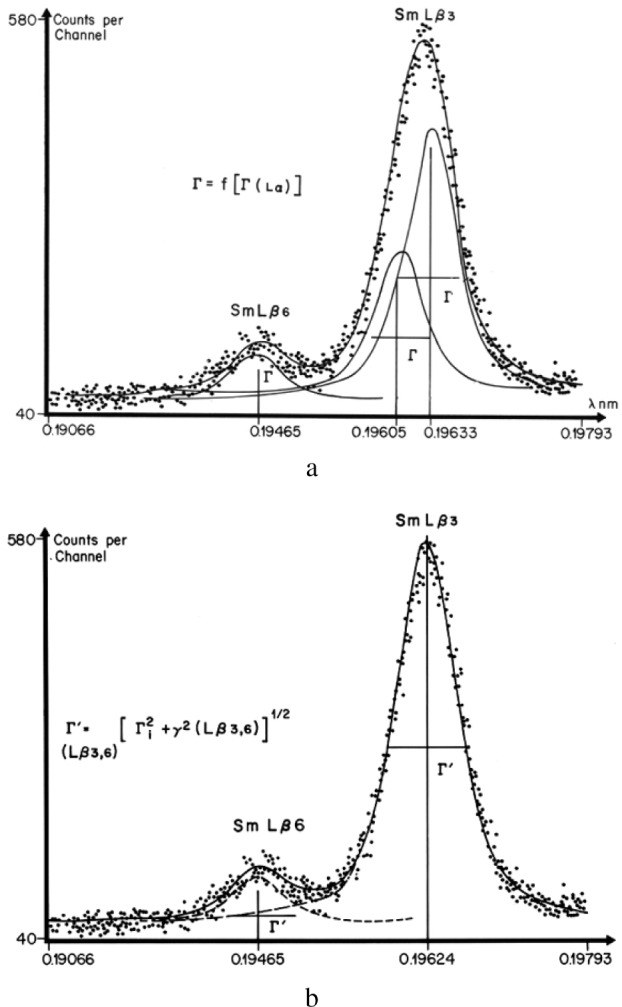
Results of fits to the SmLβ_3,6_ peaks analyzed with a LiF monochromator. (a) using the FWHM value derived from a calibration curve of widths for Lα lines and (b) using FWHM derived from Eq. (22) accounting for the natural width and the instrumental resolution.

**Fig. 17:**
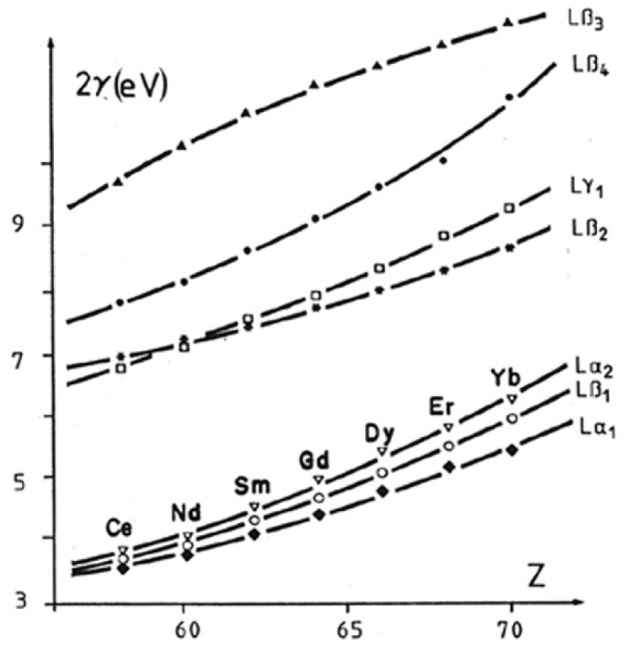
Natural widths of some L x-ray lines of the rare-earth elements according to Salem and Lee [31].

Differences in width not only exist between K, L, and M emission lines of similar energy, but also exist between the multiple lines of the L series of the same element. Neglecting the contribution of the natural width to the observed width may lead to the detection of artifacts as illustrated for SmLβ_3_ emission line [1]. Peak shape analysis of the SmLβ_3_ using the width as a variable in the fit leads to a larger width of the peak than that of the SmLα peak. It was thus tempting to identify the Lβ_3_ peak broadening as the result of two unresolved lines as shown in Fig. 16a. For these calculations, the width of the two components were set at the value derived from the calibration curve *Γ*_i_ = *f*[*Γ*(Lα)]. Such decomposition led to the detection of a spurious peak since the Lβ_3_ is a single line as shown in Fig. 16b when the width of the peak was set to that calculated by Eq. (22), including the instrumental resolution of the monochromator and the natural width of the Lβ_3_ line. As shown in Fig. 17, the natural widths of the Lβ_3_ lines for the rare-earth elements are approximately twice those of the Lα line of the same element [31]. These differences are responsible for the measured widths derived from the peak shape analysis of the L lines of the rare-earth elements when the width of each component is kept as a variable in the fitting procedure.

### 4.3 Relative Intensities

**Fig. 18:**
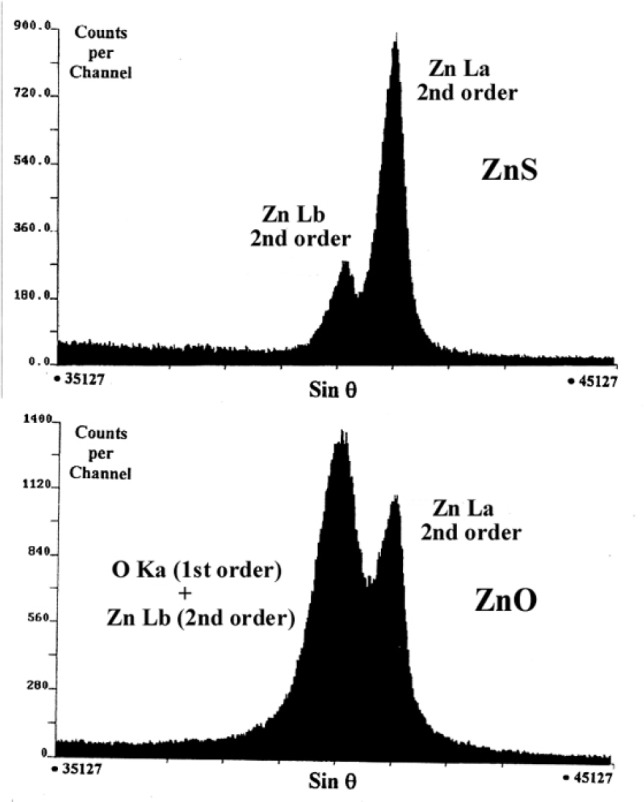
Interferences between the O Ka (first order reflection) and ZnLα,β emission bands (second order reflection) measured from a ZnO specimen using a W/Si monochromator.

Data in Fig. 18 correspond to a ZnO matrix showing interference between the fundamental OKα emission band and the second order reflection of the ZnLα,β emission bands analyzed with a W/Si multilayer structure as monochromator.

The positions, the relative intensities ZnLα,β and the intensities of the high energy satellites to the Lα and Lβ lines depend on the chemical environment and these parameters must be determined and used as coupled variables in the fit in order to obtain an accurate intensity of the OKα band which is interfering with the ZnLα,β second order reflection.

**Fig. 19:**
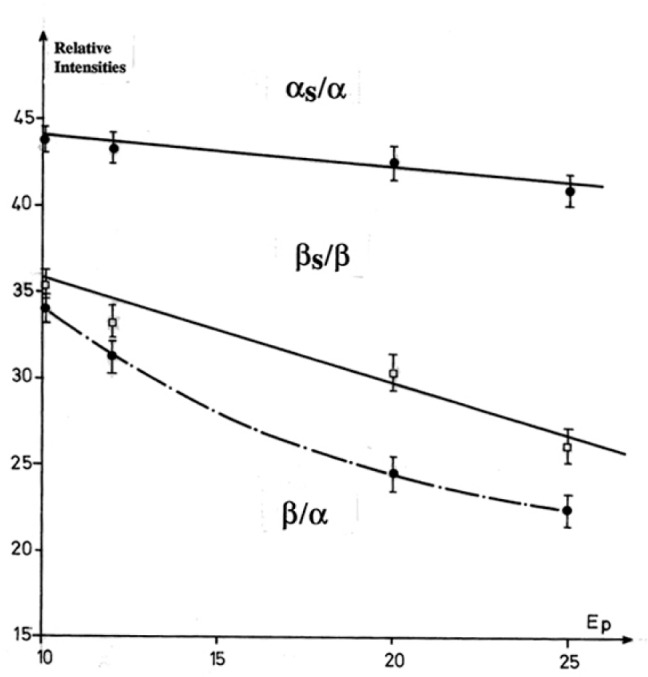
Parameters describing the ZnLα,β emission bands from a ZnO specimen measured with a TAP monochromator

To demonstrate this procedure, the fundamental ZnLα,β emission bands for the ZnO matrix were analyzed with a TAP monochromator by varying the incident energy from 10 keV to 25 keV. The energy separation distances between the ZnLα,β diagram bands and their high energy satellites remained constant. The relative intensity ratios αs/α, βs/β, and α/β as function of the incident energy are shown in Fig. 19. The theoretical separation distance [ZnLβ–ZnLα] for the diagram lines, and the intensity ratios αs/α, βs/β, and β/α derived from Fig. 19 were used as coupled variables in the fitting function describing the interfering OKα–ZnLα,β emission bands analyzed with the W/Si LMS.

**Fig. 20:**
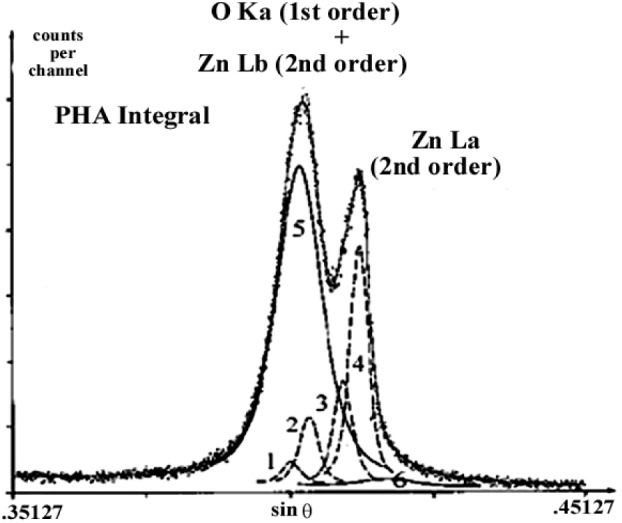
Results of fit to the ZnLα,β and OKα emission bands for a ZnO specimen analyzed with a W/Si multilayer structure as monochromator. The ZnLα,β diagram lines were described by pseudo-Voigt profiles (2)(4) with high energy pseudo-Voigt profiles (1)(3) associated with satellite bands. The parameters describing the α, β, α_s_ and β_s_ components were set as coupled variables according to the data shown in Fig. 19. All parameters in the pseudo-Voigt profiles (5)(6) describing the OKα peak shape were kept as independent variables. The digital spectra were acquired using the integral operating mode of the PHA.

**Fig. 21:**
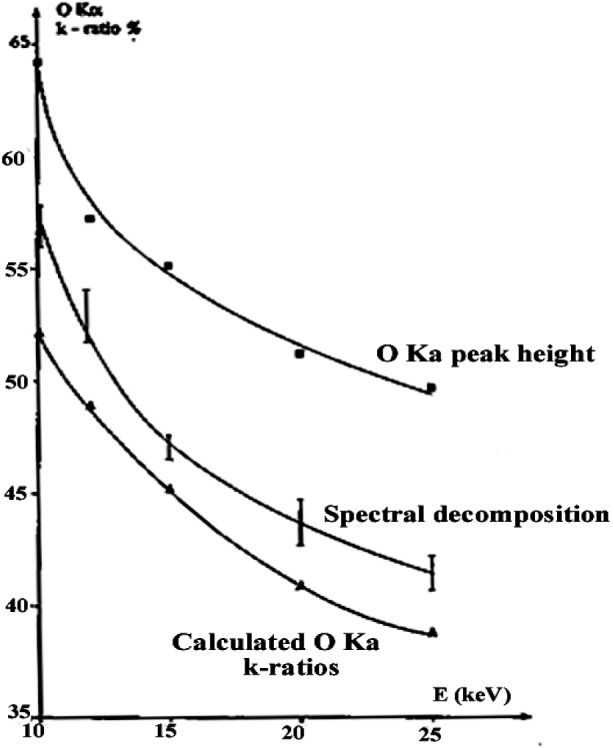
Variations as a function of the incident energy of the OKα intensity ratios for a ZnO specimen with respect to an Fe_2_O_3_ reference specimen. All spectra were acquired using the integral operating mode of the PHA.

Results of the curve fitting are shown in Fig. 20. As expected because of the peak interferences, the OKα *k*-ratios for the ZnO specimen analyzed with the Fe_2_O_3_ standard were in a better agreement with the calculated data when the peak decomposition procedure was used instead of the peak height measurement as illustrated in Fig. 21. A small deviation between the measured and calculated *k*-ratios was still observed. This deviation may result from either uncertainties in some parameters (mass absorption coefficient, ionization cross-sections, etc.) or from differences in the intrinsic properties of the analyzed specimens.

### 4.4 Peak Shape Modifications Related to the Presence of Absorption Edges

#### 4.4.1 FeLα,β Emission Bands From Pure Iron

**Fig. 22:**
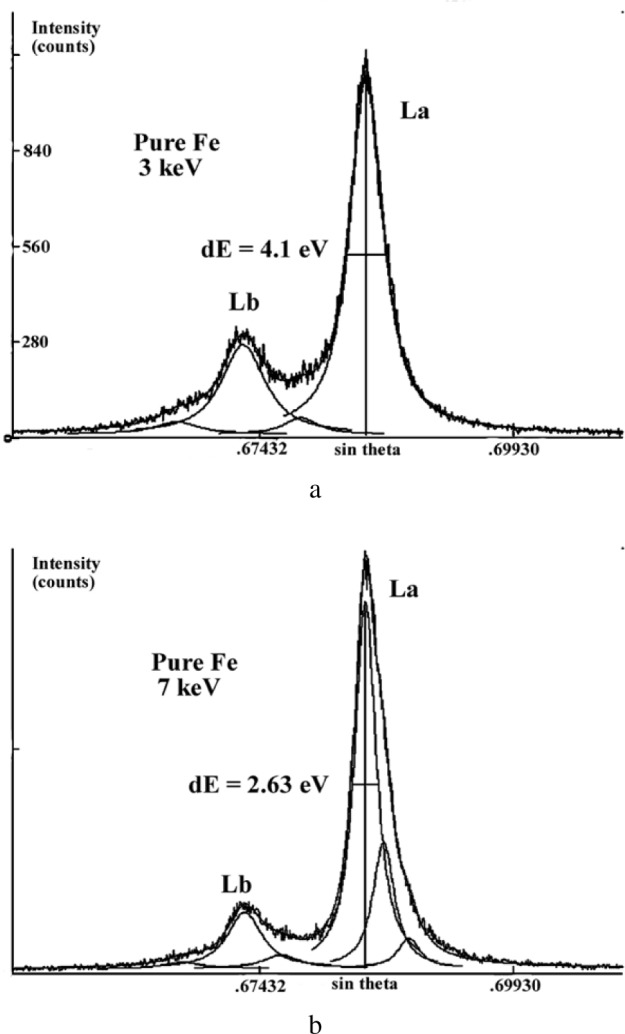
FeLα,β spectra for pure Fe measured with a TAP monochromator and (a) a 3 keV incident energy and (b) a 7 keV incident energy successively. Spectral decomposition into pseudo-Voigt profiles are also show on these figures.

The shape of the FeLα,β emission spectra also depends on the electron incident energy as illustrated in Fig. 22. The spectra were measured with a TAP monochromator, the incident energy was 3 keV and 7 keV. The width at half-maximum (FWHM) increases as the incident energy is decreased from 7 keV to 3 keV. However, the FeLα to the FeLβ intensity ratio is higher for a 3 keV incident energy compared with the ratio at 7 keV energy.

The emission spectrum measured with a 3 keV energy was processed using Eq. [17] as a fitting function and assuming a linear variation of the intensity of the continuous emission within the analyzed wavelength domain (Fig. 22a). The low energy side (long wavelength side) of the FeLα peak was correctly described by a pseudo-Voigt profile with *C*_g_ = 0.1 expressing a near Lorentzian shape of the peak. In order to obtain a satisfactory quality of fit to the Lα peak a second pseudo-Voigt component of weak amplitude was added on the high energy side (short wavelength side) of the peak. Similarly, two pseudo-Voigt profiles with *C*_g_ = 0.1 were used to described the FeLβ peak. These high energy components probably correspond to satellite lines resulting from shake-off or Coster-Kronig mechanisms. The results of fit led to a 4.0 eV FWHM for the FeLα peak consistent with the 3.7 eV FWHM value reported by Bonnelle [32]. The near Lorentzian peak shape of the spectrum measured with a 3 keV incident energy indicates that the calculated FWHM values are probably close to those of the natural Lorentzian width and that for the analyzed wavelength region, the TAP monochromator has a small contribution to the observed peak shape. This result is also supported by data previously reported by Rémond et al. [5] studying the FeLα,β emission bands measured with a 15 keV incident energy. For that incident energy, the FeKα_1,2_ ninth order reflection are detected between the FeLβ and the FeLα peaks. The FWHM of the FeKα_1,2_ peaks were found to be 3.4 eV and 3.8 eV, respectively. These values are similar to intrinsic widths reported by Salem and Lee [31], supporting the weak instrumental broadening of the monochromator in the analyzed region.

The FeLα,β emission bands measured with a 7 keV incident energy have a narrower FWHM values than for the spectrum measured with the 3 keV energy as shown in Fig. 22b for the FeLα peak of the pure Fe specimen. Consequently, the FeLα from pure Fe is no longer described by a single pseudo-Voigt profile. Two pseudo-Voigt profiles must be added to the low energy side of the major component but these two offsets have no physical meaning since for a pure metal no low energy satellites are expected with significant amplitudes. The departure from symmetry of the FeLα emission band results from a differential absorption for the high and low energy sides of the peak because the L_3_ absorption edge intercepts the high energy side of the peak. A similar situation is encountered for the case of the L_2_ absorption edge which intercepts the high energy side of the Lβ peak.

**Fig. 23:**
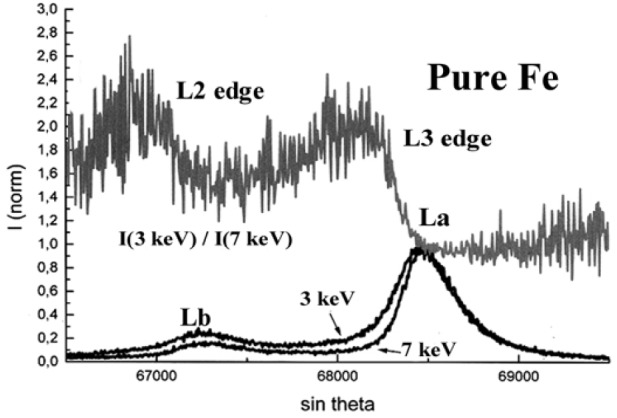
Absorption correction factor within the wavelength region containing the FeLα,β emission bands from pure Fe.

**Fig. 24:**
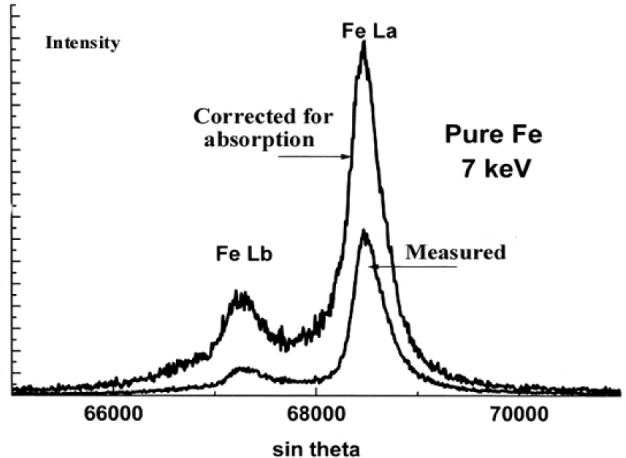
Reconstructed generated FeLα,β emission spectra in pure Fe at 7 keV incident energy.

**Fig. 25:**
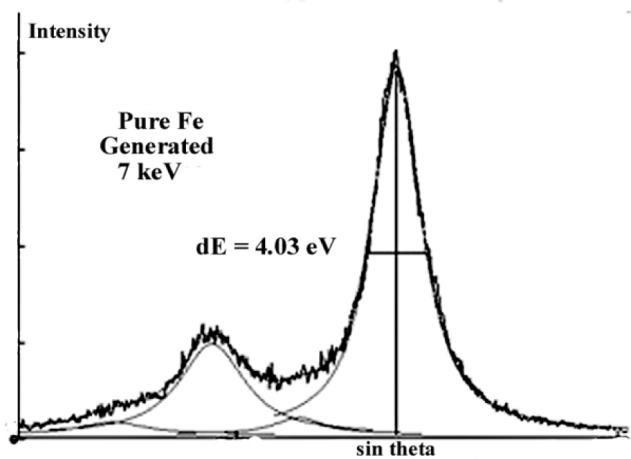
Spectral decomposition of the generated FeLα,β emission spectra at 7 keV incident energy for pure Fe.

The experimental spectra measured with a 7 keV incident energy were corrected for self-absorption using the approach described previously which consists in dividing the normalized spectra measured at 3 keV and 7 keV successively resulting in the *g*(*χ*) curve as shown in Fig. 23. Each channel of the experimental spectrum measured with the 7 keV incident energy is divided by the value of the *g*(*χ*) curve giving an experimental spectrum whose the shape is corrected for the presence of the absorption edge in the analyzed region. The corrected spectrum is then multiplied channel by channel by the scaling factor in Eq. [21] where *f*(*χ*_0_) is calculated using the tabulated mass absorption coefficient of the analyzed elements for the monochromatic FeLα line. The reconstructed FeLα,β spectra at 7 keV incident energy is shown in Fig. 24 for the pure iron specimen and was decomposed into pseudo-Voigt profiles, as shown in Fig. 25.

#### 4.4.2 FeLα,β Emission Bands From Iron Oxides

Synthetic Fe_0.94_O (referred below as FeO) and natural Fe_2_O_3_ specimens were analyzed according to the experimental conditions mentioned above. As for pure iron, the shape and the peak maximum position depend on the incident energy. The shift of the FeLα,β peak position is larger for the iron oxides than for the pure iron specimen. As an example, the shift for pure Fe was 0.18 eV when the incident energy was increased from 3 keV to 7 keV but was 0.8 eV for the FeO specimen.

**Fig. 26:**
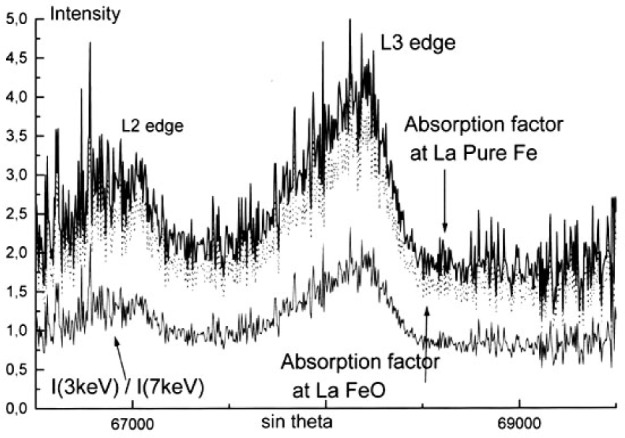
Absorption correction factors for FeLα in FeO within the wavelength region containing the FeL emission bands.

The correction curve *g*(*χ*) derived from the normalized spectra measured at 3 keV and 7 keV successively is shown in Fig. 26. In order to obtain the absorption correction factor for each analyzed channel, the *g*(*χ*) curve must be multiplied by the scaling factor (Eq. [21]) for the excitation conditions used.

**Fig. 27:**
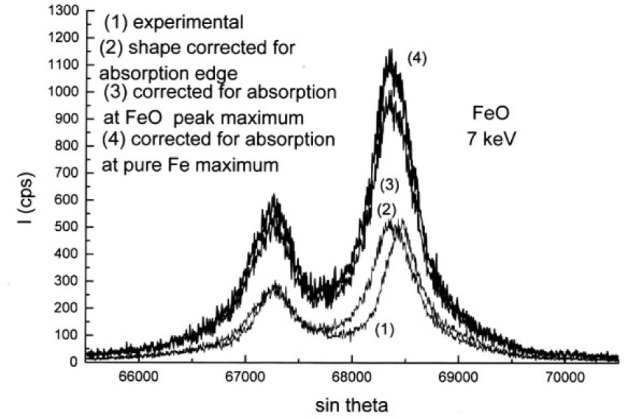
FeLα,β emission spectrum corrected for absorption from an FeO specimen measured with a 7 keV incident energy.

In order to calculate the *f*(*χ*) factor, the mass absorption coefficient associated to the maximum position of the emission bands for the analyzed specimens must be known. In practice, only the mass absorption coefficient for FeLα at the diagram FeLα emission position for pure Fe is obtained from data Tables. For the iron oxides, the absorption coefficient is calculated by weighting the mass absorption coefficients for FeLα by the mass concentrations of iron and oxygen in the analyzed iron oxide specimens. Figure 26 illustrates the variation of the correction factors within the analyzed wavelength domain when the absorption factor for the monochromatic FeLα line is applied to the maximum position of the pure Fe or the iron oxide specimens analyzed with a 7 keV incident energy. The corresponding corrected spectra representing the generated intensity of the FeLα,β emission bands are shown in Fig. 27 for the FeO specimen. Similar results are obtained for the Fe_2_O_3_ specimen.

**Fig. 28:**
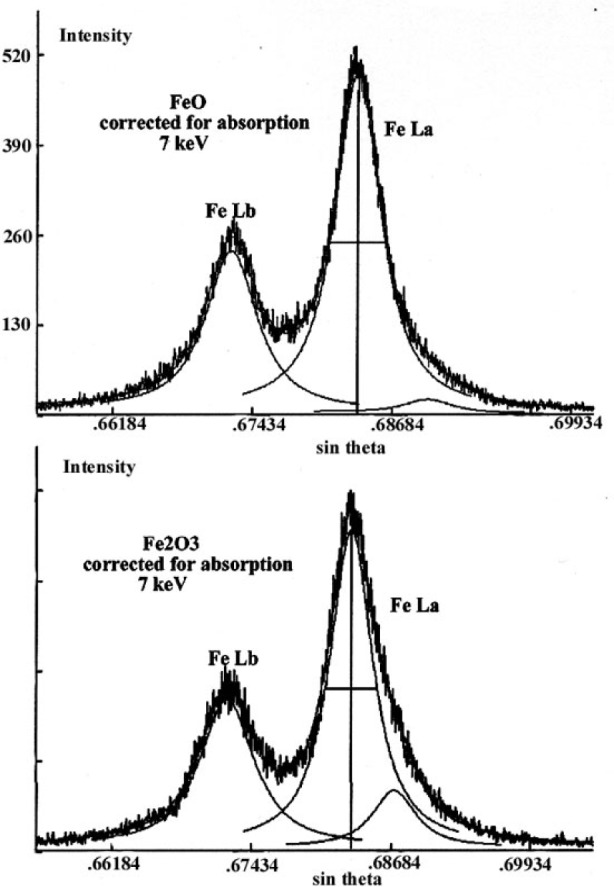
Decomposition into pseudo-Voigt profiles of spectra corrected for self-absorption in FeO and Fe_2_O_3_ specimens analyzed with a 7 keV incident energy.

Spectra corrected for self-absorption were decomposed into the sum of pseudo-Voigt profiles as shown in Fig. 28. Two pseudo-Voigt profiles of equal width were used to describe the Lα emission band. The separation distance between the major diagram peak and the low energy band as well as the relative intensity of the two features are different for the FeO and the Fe_2_O_3_ specimens as shown in Table 1. The diagram peaks are accompanied by a low energy band probably resulting from bonding states and possibly with a contribution of radiative Auger emission, which can be measured owing to the energy resolution and sensitivity of a WDS as illustrated by Takahashi et al. [34].

The FeLα intensity from the FeO specimen was expressed in terms of mass concentration using the Fe_2_O_3_ emission spectrum as a reference. The total area of the experimental FeLα band, i.e., the diagram band and the low energy band, was used for quantitative analysis and the experimental concentration, *k*, was corrected for absorption according to the usual procedure used in EPMA analysis. In a second experiment, the emission spectra measured with a 7 keV incident energy were first corrected for self-absorption before to determine the total peak areas. Thus, the intensities derived from the fits represent the generated intensities. The ratio of the FeLα intensity measured from the corrected spectra for FeO and Fe_2_O_3_,respectively, directly gives the concentration of iron. Quantitative results are shown in Table 2. Correcting the spectra for non-uniform *f*(*χ*) absorption correction factor within the FeLα emission bands leads to an improvement of the quantitative results compared with those derived from the conventional approach where only the absorption factor associated with the maximum emission of the analyzed x-ray emission is applied.

The use of the total area of the emission band as intensity of the analyzed soft x-ray emission still remains questionable. The low energy band associated with the diagram emission band involves transitions from valence electrons, some of them originating from oxygen states. From this study it is not possible to conclude whether the total peak area or that of the different spectral components of a complex x-ray emission spectrum must be used as intensity measurement in the quantitative analysis. In the present study, the low energy band to the diagram emission band was simply described by a pseudo-Voigt profile. This band is probably complex and the pseudo-Voigt profile only represents the envelope of many features. Improvement in the fitting procedure will be performed when theoretical models of the radiative mechanisms will be undertaken. Further study is required on compounds where the same element is present in different valence states in order to account for the differential absorption effect.

The uncertainty in the quantitative data also results from the choice of the mass absorption coefficients to be used for the calculation of the *f*(*χ*) absorption correction factor. However, the use of mass absorption coefficients given for pure elements remains questionable since, owing to the difference in the electronic structure, the mass absorption coefficients for FeLα for pure iron and the iron oxides are expected to be different.

## 5. Conclusion

The energy resolution of monochromators used with the EPMA usually have a sufficient resolution to observe non-diagram bands occurring either on the short or long wavelength side of the main diagram peak. The observed line shape is the convolution of the natural physical width with the instrumental response function. For x-ray emission lines occurring at low Bragg angles, broadening and asymmetry of the measured x-ray peaks are observed. The shape of an atomic x-ray peak (resulting from transitions only involving core level electrons) or of an x-ray emission band (involving valence electrons) has been described by pseudo-Voigt profiles in a least-squares fitting analysis of experimental WDS spectra which includes analytical description of the observed emission.

Owing to the large number of variables involved in the analytical description of complex observed x-ray emission bands, physical constraints must be applied, as illustrated with the line widths, the relative intensities of the spectroscopic components, and the distortion of the peak profiles, resulting from either instrumental factors or from the presence of absorption edges in the analyzed wavelength domain.

In order to add physical constraints in the fitting procedure, a database is particularly important to develop for complex soft x-ray emission spectra. However, it is apparent from this review on WDS peak shape analysis that for soft x-ray, the correction for self-absorption in the entire wavelength region containing the analyzed emission bands represent an improvement in quantitative analysis using soft x-ray emission peaks.

## Figures and Tables

**Table 1:** Spectral decomposition of the FeLα emission corrected for self-absorption (7 keV incident energy)

	Lα Diagram band		Low energy band	
Specimen	Position (eV)	Width (eV)	Position (eV)	Relative intensity(%)
FeO	704.3	5.4	697.7	4.0
Fe_2_O_3_	704.7	4.8	700.7	15.1

**Table 2:** Quantitative analysis of FeO using Fe_2_O_3_ as standard

Ep (keV)	Spectra	*C*	Δ*C/C* %
3	Measured	0.760	–1.3
7	Measured	0.592	–23
7	Corrected for edges	0.757	–1.7
